# Thyroid Function at Age Fifty After Prenatal Famine Exposure in the Dutch Famine Birth Cohort

**DOI:** 10.3389/fendo.2022.836245

**Published:** 2022-06-30

**Authors:** Sarai M. Keestra, Irina Motoc, Anita C.J. Ravelli, Tessa J. Roseboom, Martijn J.J. Finken

**Affiliations:** ^1^Department of Epidemiology & Data Science, Amsterdam University Medical Centre (UMC), University of Amsterdam, Amsterdam, Netherlands; ^2^Department of Reproductive Medicine, Amsterdam University Medical Centre (UMC), Vrije Universiteit Amsterdam, Amsterdam, Netherlands; ^3^Amsterdam Reproduction & Development Research Institute, Amsterdam, Netherlands; ^4^Department of Paediatric Endocrinology, Amsterdam University Medical Centre (UMC), Vrije Universiteit Amsterdam, Emma Children’s Hospital, Amsterdam, Netherlands; ^5^Department of Medical Informatics, Amsterdam University Medical Centre (UMC), University of Amsterdam, Amsterdam, Netherlands; ^6^Department of Obstetrics & Gynaecology, Amsterdam University Medical Centre (UMC), University of Amsterdam, Amsterdam, Netherlands

**Keywords:** famine, thyroid, DOHaD (Developmental origins of health and disease), TSH (thyroid stimulating hormone), thyroxine (FT4)

## Abstract

**Background:**

Early-life exposures during gestation may permanently alter thyroid physiology and health in adulthood. We investigated whether exposure to the Dutch Famine (1944-1945) in late, mid, or early gestation influences thyroid function (i.e., incidence of thyroid disease, thyroid autoantibodies, thyroid stimulating hormone (TSH), and free thyroxine (FT4) levels) in adulthood. We specifically assessed whether potential effects of famine differed for men and women.

**Methods:**

This study includes 910 men and women born as term singletons in the Wilhelmina Gasthuis in Amsterdam, the Netherlands, shortly before, during, or after the Dutch Famine. We evaluated medical histories for previous diagnosis or current treatment for thyroid dysfunction. At age 50 blood samples were drawn from 728 individuals for tests of thyroid function. We studied the prevalence of overt hypo- and hyperthyroidism and thyroid autoimmunity using medical histories, and measurements of TSH, FT4, anti-TPO and anti-TG, comparing participants exposed to famine at different pregnancy trimesters or born before or conceived after the famine. Additionally, we studied associations of TSH and FT4 levels with *in utero* famine exposure in a subsample of men and women free of thyroid disease that were exposed in late, mid, or early gestation.

**Results:**

There were no differences in thyroid dysfunction diagnosis or current treatment between participants at age 50 years who been exposed to famine during different periods of gestation and those born before or conceived after. There was no association between famine exposure and overt hypo- or hyperthyroidism or thyroid autoantibody positivity. Women who had been exposed to famine in mid gestation had slightly lower TSH levels than women who had not been exposed to famine prenatally (b=-0.06; 95%; CI=[-0.11,-0.02]; p<0.01). No differences in TSH levels were observed in men, and no differences in FT4 levels were observed in men or women.

**Conclusions:**

There are no differences in adult thyroid disease at age 50 years according to prenatal famine exposure. However, the lower TSH levels in women exposed to famine in the second trimester suggest that there may be sex-specific effects of famine exposure during a critical period of thyroid development on hypothalamic-pituitary-thyroid axis regulation in adulthood.

## Introduction

Thyroid disease is among the most common endocrine disorders, yet little is known about the effect of adverse conditions during early development on thyroid function regulation in adulthood (1). Thyroid dysfunction groups with obesity-related non-communicable diseases such as cardiovascular disease, type 2 diabetes mellitus, and polycystic ovary syndrome (2–4). Slightly elevated TSH levels are frequently found in individuals with metabolic syndrome, which refers to the clustering of visceral adiposity, insulin resistance, hypertension, and dyslipidaemia (5, 6). These metabolic conditions have all previously been associated with prenatal undernutrition or foetal growth restriction followed by affluence (7, 8). The framework of Development Origins of Health and Disease (DOHaD) offers the perspective that there are critical periods of development when physiological systems are highly flexible and environmental conditions play an important role in the long-term programming of human health (9–11). During this window of developmental plasticity endocrine, nutritional, and stress-related cues may alter an individual’s physiological make-up and affect the epigenetic programming of key metabolic pathways (9, 11). This might include the hypothalamic-pituitary-thyroid (HPT) axis, which through a negative feedback loop *via* the anterior pituitary’s release of thyroid stimulating hormone (TSH), regulates the production of thyroid hormones that contribute to the regulation of metabolism, reproduction, and growth (1). Yet, the importance of timing of severe malnutrition at different pregnancy trimesters on adult thyroid function has not been studied previously in humans, despite possible implications for the occurrence of metabolic syndrome.

Research from the Netherlands Twin Register suggests that between 22-27% of variance in thyroxine (T4) in neonates is due to shared environment in the womb and a further 33-47% due to environmental factors (12). This suggests a role for foetal environment in the development of HPT axis setpoints (12). The thyroid becomes active after the 12th week of gestation (1), and during the second trimester of gestation undergoes a large increase in size (13). Famine exposure during critical development could disrupt these processes, leading to slightly altered thyroid physiology in later life. Studies in rats have found that undernutrition during gestation starting at the day of mating predisposed towards a pattern of elevated TSH and lower plasma free T4 in adulthood (14). Conversely, a study in sheep found that undernutrition in the last six weeks of pregnancy followed by overnutrition postnatally predisposed towards adult hyperthyroidism (15). Similarly, in rats protein-restricted maternal diets during lactation led to lower TSH levels and hyperthyroidism in adult offspring (16). In humans exposed to the Chinese famine of 1959-1961, one study found that individuals exposed *in utero* had significantly higher TSH levels (within the normal range) in adulthood, in contrast to those born before or after the famine (17). Another study on individuals exposed to the Chinese famine in early development did not find altered thyroid function in individuals exposed *in utero*, instead finding lower free T4 and higher TSH levels in adults postnatally exposed to the famine (18). Overall, these studies suggests that exposure to famine during early life may affect thyroid function depending on the timing of the exposure; specifically, studies on the Chinese famine suggest that exposure to famine conditions from early pregnancy onwards may be associated with a subtle downregulation of the HPT axis and higher TSH levels in adulthood (17, 18). Amongst famine-exposed Chinese, there was not a higher incidence of overt thyroid dysfunction (17, 18), suggesting that in humans the overall effect of undernutrition in early life on thyroid function regulation is subtle.

In contrast to the Chinese famine, which lasted between 1959 and 1961 (17, 18), the Dutch famine of 1944-1945 lasted for a shorter period of time, had an abrupt beginning and end in an otherwise affluent and well-nourished population (19). Because of its well-defined exposure periods and the conservation of the original birth records of the main maternity hospital in Amsterdam (19), the Dutch Famine Birth Cohort (DFBC) offers a unique opportunity to consider whether timing of famine exposure during different pregnancy trimesters may lead to differences in adult thyroid function. The DFBC is a prospective birth cohort that looks at the health of individuals born shortly before, during, or after the Dutch Famine of 1944-1945, also known as the ‘Hunger Winter’, in the Wilhelmina Gasthuis in Amsterdam (19). During this period, a ban of food transport into the occupied Western region of the Netherlands, as a retaliation against the local population for a railroad strike in support of the Allied forces, led to an acute famine, which in combination with a harsh winter resulted in extreme nutritional deprivation in an otherwise affluent population (19, 20). Daily food rations, which were recorded in detail since 1941, declined to only 500-600 calories a day, which abruptly ended when the Netherlands was liberated on the 5^th^ of May 1945 (19, 20). Nutritional status afterwards rapidly improved and by June 1945 food rations included 2,130 calories a day (21). Thus, the events of the 1944-1945 winter offer a historic opportunity, as a time-limited, well characterised ‘natural experiment’ to understand the long-term effects of undernutrition during early development followed by more prosperous conditions during child- and adulthood in post-World War Netherlands on thyroid (dys)function in later life. Making use of thyroid function tests at age 50 in combination with extensive medical histories, the DFBC offers the opportunity to further explore the long-term effect of the timing of prenatal famine exposure during pregnancy on the programming of the HPT axis. This study aims to investigate whether overt hypo- and hyperthyroidism is more common amongst those exposed to the famine during gestation, and whether there is any difference in normal variation in thyroid function (TSH and FT4) in men and women depending on the timing of exposure to famine *in utero*.

## Methods

### Study Population

This study is based on data collected in 1996-1997, when the participants were 50 years old, by the DFBC study team at the Academisch Medisch Centrum (AMC), now the Amsterdam University Medical Center (UMC). ﻿The study received ethical approval from the Medical-Ethical Committee of the Academic Medical Centre of the University of Amsterdam. The study design and selection procedures of the DFBC have extensively been described elsewhere (19). In short, all singleton babies born at term in the Wilhelmina Gasthuis in Amsterdam between the 1^st^ of November 1943 and 28^th^ of February 1947 were eligible for inclusion in (n=5,425) (22, 23). The cohort thus included 1,380 individuals that were exposed to famine *in utero* based on their birth date (1 November 1944 and 28 February 1946). Additionally, a random sample of babies born the year before or conceived after this pre-defined period were selected. Exclusion criteria included premature birth (*i.e.*, gestational age <37 weeks), individuals with missing medical records, or multiple pregnancies. Of the 2,414 liveborn singletons it was possible to trace 2,155 individuals (89.2%) using Amsterdam’s population registry (24). 910 Individuals were visited at home and interviewed by the research team. Medical histories were recorded, including any previous diagnosis of thyroid dysfunction and current treatment of thyroid disease.

Ultimately 741 individuals were included in the first clinical data collection wave at age 50 and attended the follow-up clinic at the AMC. Exclusion criteria at this stage included death, migration, and inability to attend the clinic in Amsterdam (22). Of these participants that attended the follow-up clinic, 728 individuals provided blood samples from which thyroid function (at least TSH as well as free T4) could be determined. Participants are divided up into the following groups based on birth date as an indication of trimester of famine exposure (19, 22): i) those born shortly before the famine (born 11/1943-1/1944); ii) those mainly exposed in late gestation (born 1/1945-4/1945); iii) those mainly exposed in mid gestation (born 4/1945-8/1945); iv) those mainly exposed in early gestation (born 8/1945-12/1945); v) those conceived after famine exposure (12/1945-2/1947). Of the 728 participants, 291 were *in utero* whilst being exposed to the famine, whereas another 437 were born shortly before or after the famine.

### Laboratory Analyses

Blood samples were drawn in the morning. Thyroid function tests included assessment of thyroid stimulating hormone (TSH), free thyroxine (FT4), and antibodies against thyroid peroxidase (TPO) and thyroglobulin (TG). TSH was measured using an immunofluorimetric assay (IFMA) by Delfia, Wallac - Perkin Elmer (cut-off: 0,4 – 4,0 mE/L). FT4 was measured with a fluorescent immunoassay by Delfia, Wallac - Perkin Elmer (cut-off: 10 – 23 pmol/L). Anti-TPO and anti-TG were measured using radioimmunoassay by Brahms and in house of the AMC respectively. For the autoantibody positivity the cut-off for positivity was set at >100 kU/L. These thyroid function tests were analysed in batch in the endocrinology laboratory of the Amsterdam university hospital in 1997 shortly after collection of the data in 1994-1996.

### Definitions of Thyroid Disease

Using medical histories collected during interviews at home (n=910), we identified anyone with a previous diagnosis of thyroid dysfunction, being treated for a thyroid condition, or taking medication for hypothyroidism. Based on the thyroid function tests in combination with the medical histories of the participants for whom blood samples were available (n=728), we report of rates of overt hypo- and hyperthyroidism in early, mid, and late gestation exposure groups and those born before or after the famine occurred. For the purpose of this study, we defined overt hypothyroidism as i) previous diagnosis of hypothyroidism (either of autoimmune origin or as a result of post-thyroidectomy) as self-reported in the medical history, ii) taking thyroid medication (prescribed as eltroxin, euthyrox, thyrax, thyrofix, tirosint, or as generic levothyroxine in the Netherlands), iii) a TSH >10 mE/L or a combination of TSH>4mE/L and FT4<10 pmol/L at the time of blood collection. Hyperthyroidism was defined as i) current diagnosis of hyperthyroidism (due to Graves’ disease or another reason), ii) TSH levels <0.4 mE/L in combination with FT4 >23 pmol/L. Anti-TPO and anti-TG positivity were used to determine thyroid autoimmunity.

### Statistical Analyses

Firstly, we reported on sample characteristics according to timing of famine exposure *in utero*, differentiating between early, late, and mid gestation, as well as those born before or conceived after the famine in the complete cohort of 910 individuals. Using thyroid function tests in 728 individuals from which blood samples were taken, we then assessed significance of any differences in overt thyroid dysfunction or thyroid autoantibodies according to timing of famine exposure and sex. We used binary logistic regression models to estimate the adjusted odds ratios (OR) with 95% confidence interval (95%CI).

Excluding participants with overt hypo- or hyperthyroidism based on thyroid function tests, previous diagnosis of thyroid dysfunction as recorded in the medical history, currently taking thyroid medication or other medication that may affect thyroid function such as amiodarone, we report the sex- and trimester-specific effect of famine exposure. One participant with an abnormally high FT4 level (>50 mE/L) but normal TSH level was also excluded from this subsample as this measurement was attributed to a measurement artefact. This created a subsample free from overt thyroid disease with thyroid function tests in the normal range. Next, TSH levels were log-transformed and estimates were back transformed, to ease interpretation. The following formulas were used for the log-transformation and the back transformation: ***Log_10_(1+TSH)*** and ***Exp(TSH)-1.*** Using a Two Sample T-test, we first determined whether there were any significant differences in TSH or FT4 levels between individuals who were born shortly before or conceived shortly after the famine, pooling these into one group of individuals not exposed to famine during gestation when no significant difference was found. We then used linear regression models to investigate the interaction between sex and famine exposure. In model 1 the association between timing of famine exposure and TSH and FT4 levels were assessed by entering exposure groups in the model as dummies and setting the group that was not exposed to famine *in utero* as the reference category. Model 2 was besides famine exposure further adjusted for sex. In model 3, interaction terms between sex and famine exposure groups (early, mid, late) were added. In case of a sex effect, where an interaction was deemed significant for p-values smaller than 0.1, the sample was sex-stratified.

The following possible confounders were selected by examining their (bivariate) association with TSH and FT4 levels, at a significance level of p<0.1 for either TSH or FT4. Smoking was defined as those who reported at the time of the interview that they had been smoking longer than 1 month (23). Menopausal status was calculated as the last menstrual period being more than 12 months in the past from the date of the study interview. Socioeconomic status was based on the International Socio-Economic Index (ISEI-92) which is a scale of socioeconomic status between 16 (low) and 87 (high) and was constructed from the highest socioeconomic status between study participant and their partner (25). All statistical analyses were conducted in RStudio (version 1.3.9590).

## Results

Of the 910 participants included, 4.9% had previously been diagnosed with a thyroid dysfunction, and 1.8% were currently under treatment for the condition (**Table 1**). Based on the blood samples taken from 728 participants, 2.3% had thyroid autoantibodies (anti-TPO or anti-TG) above the clinical threshold. The incidence of overt hypothyroidism in this sample based on thyroid function tests and medical histories was 4.4%, whereas the incidence of overt hyperthyroidism was 2.2% (**Table 2**). There was no significant difference in prevalence of hypothyroidism, hyperthyroidism, or thyroid antibodies between early, mid, and late gestation famine exposed groups and those born before or after the famine. However, the odds of hypothyroidism were greater amongst women than men (OR=2.16, 95%CI [1.04,4.84], p<0.05), as were the odds of hyperthyroidism (OR= 4.24, 95%CI [1.35-18.60], p<0.01). Furthermore, more women than men had thyroid autoantibody levels (anti-TPO and/or anti-TG) above the clinical threshold (antibody positivity OR= 4.54, 95%CI [1.47-19.81], p<0.02).

**Table 1:** Mean descriptives of the DFBC clinical visit cohort that had thyroid function tests taken at age 50 years old, according to timing of famine exposure in early life (n=910).

Timing of gestation exposure to famine						
Prenatal famine exposure	Born before (n=264)	Late gestation exposure (n=140)	Mid gestation exposure (n=137)	Early gestation exposure (n=87)	Conceived after (n=282)	All(n=910)
Women (%)	50.8	52.9	61.3	58.6	50.4	53.3
Menopausal women (%)	14.2	13.5	16.7	21.6	9.9	14.0
Height [cm]	170.6	170.1	168.4	170.4	170.3	170.1
Weight [kg]	78.8	78.2	76.5	80.2	80.4	79.0
BMI [kg/m2]	27.0	27.0	26.9	27.6	27.7	27.3
Socioeconomic status [ISEI-92]	46.2	49.7	48.3	47.6	47.8	47.7
Current smokers (%)	37.5	37.1	32.1	47.1	35.1	36.8
Previously diagnosed thyroid dysfunction, by medical doctor (%)	4.2	3.6	4.4	6.9	6.0	4.9
Currently being treated for thyroid dysfunction (%)	1.5	1.4	0	2.3	2.8	1.8
Taking thyroid medication (%)	0.4	0	0	0	1.8	0.7

**Table 2:** Prevalence and odds ratio of thyroid dysfunction in the DFBC depending on sex and famine exposure group (born before, late, mid, or early gestation, or conceived after) as well as sex in a subsample for which thyroid function tests were available (n=728).

		Timing of gestational exposure to famine					Sex			
		Born before (n=209)	Late gestation exposure (n=119)	Mid gestation exposure (n=106)	Early gestation exposure (n=66)	Conceived after (n=228)	Women (n=373)	Men (n=355)	All (n=728)
Overt hypothyroidism	% (n)	6.2 (12)	3.4 (4)	6.6 (7)	3.0 (2)	3.1 (7)	5.9 (22)	2.8 (10)	4.4 (32)
	OR [95%CI]	1.92 [0.76, 5.26]	1.10 [0.28, 3.71]	2.23 [0.74, 6.69]	0.99 [0.14, 4.20]	–	**2.16** **[1.04, 4.84]**	–	–
Overt hyperthyroidism	% (n)	1.4 (3)	2.5 (3)	2.8 (3)	3.0 (2)	2.6 (5)	3.5 (13)	0.9 (3)	2.2 (16)
	OR [95%CI]	0.65 [0.13, 2.68]	1.15 [0.23, 4.78]	1.30 [0.26, 5.39]	1.39 [0.20, 6.63]	–	**4.24** **[1.35, 18.60]**	–	–
Thyroid autoantibody positivity	% (n)	3.3 (7)	3.4 (4)	1.9 (2)	1.5 (1)	1.3 (3)	3.7 (14)	0.8 (3)	2.3 (17)
	OR [95%CI]	2.26 [0.72, 12.29]	1.16 [0.06, 9.26]	1.44 [0.19, 8.82]	2.63 [0.57, 13.55]	–	**4.54** **[1.47, 19.81]**	–	–

Bolded values indicate a p-value <0.05

Individuals with overt hyper- or hypothyroidism were excluded from subsequent analyses, as was anyone previously diagnosed by a medical doctor with thyroid dysfunction or currently being treated for a thyroid condition, leading to a final sample of 675 participants. We did not find significant differences between the TSH and FT4 levels of participants born before or conceived after the famine (TSH t=0.58, p=0.56; FT4 t=-0.14, p=0.89) (results not shown), therefore individuals not *in utero* during the famine were grouped to form the unexposed group. **Supplementary Table 1** shows the levels of TSH (geometric mean and interquartile range (IQR)) and FT4 (mean and standard deviation (SD)) of the early, mid, and late gestation exposed groups as well as the unexposed. **Table 3** presents models 1, 2, and 3 in the sample including both sexes and in the sex-stratified subsamples. Results of model 1 indicate that exposure group alone is not associated with TSH or FT4 levels, however sex was significantly associated with both TSH and FT4 levels (model 2). It was found in model 3 that adding sex and famine exposure as interaction terms, there were significant differences (p<0.1) in TSH within the early and mid exposed groups. Sex-stratified models revealed a significant trend in women towards lower TSH levels as a result of famine exposure in the second trimester of gestation, but not in men. Sex, smoking, and socioeconomic status were significantly associated with log-transformed TSH and FT4 levels in bivariate analyses, therefore we adjusted for these variables in the final analyses (**Supplementary Table 2**; **Table 4**). These final models were separated by sex based on the finding in bivariate analysis that famine exposure might affect TSH levels in the sexes differently. In these final models it was found that when correcting for confounding variables, mid-famine exposure was significantly associated with lower TSH levels in women but not men. There was no significant association between famine exposure and FT4 levels in either sex.

**Table 3:** Estimates of the association between *in utero* famine exposure group (late, mid, early) compared to participants that were not exposed to famine during gestation and thyroid function (TSH and FT4) tests in a thyroid disease free subsample (n=675).

		Model 1^a^ (n=675)			Model 2^b^ (n=675)				Model 3^c^ (n=675)			Model 1^d^ Men (n=341)			Model 1^e^ Women (n=334)		
		Late	Mid	Early	Late	Mid	Early	Sex	Sex*Late	Sex*Mid	Sex*Early	Late	Mid	Early	Late	Mid	Early
TSH	Estimate	0.0	-0.01	0.01	0.0	-0.02	0.01	**0.05**	-0.01	**-0.09**	-0.07	0.01	0.03	0.05	-0.01	**-0.06**	-0.02
	95% CI	[-0.03, 0.04]	[-0.04, 0.02]	[-0.03, 0.05]	[-0.03, 0.03]	[-0.05, 0.02]	[-0.03, 0.05]	**[0.03, 0.08]**	[-0.07, 0.05]	**[-0.14,** **-0.02]**	[-0.14, 0.01]	[-0.03, 0.04]	[-0.01, 0.08]	[-0.004, 0.10]	[-0.05, 0.04]	**[-0.10,** **-0.01]**	[-0.08, 0.04]
FT4	Estimate	-0.07	-0.08	-0.19	-0.04	0	-0.16	**-0.73**	0.08	0.56	0.98	-0.07	-0.31	-0.65	0.01	0.26	0.34
	95% CI	[-0.57, 0.43]	[-0.60, 0.45]	[-0.82, 0.44]	[-0.53, 0.46]	[-0.52, 0.52]	[-0.78, 0.47]	**[-1.09,** **-0.38]**	[-0.71, 1.16]	[-0.37, 1.59]	[-0.06, 2.30]	[-0.78, 0.63]	[-1.10, 0.49]	[-1.56, 0.25]	[-0.68, 0.70]	[-0.44, 0.95]	[-0.54, 1.19]

Bolded values indicate a p-value <0.05.^a^ Crude model with only famine exposure group; ^b^ model 1 also adjusted for sex. ^c^ model 1 adjusted for sex and includes an interaction term between sex and famine exposure group. ^d^ and ^e^ are a crude model with famine exposure groups only in sex-stratified sub-samples.

**Table 4:** Adjusted Estimates, 95% CIs, and p-values of TSH levels and FT4 levels in men (n=341) and women (n=334) exposed to famine *in utero* compared to those not exposed to famine during gestation.

		Women (n=334)			Men (n=341)		
		Late	Mid	Early	Late	Mid	Early
TSH	Adjusted Estimate	-0.01	**-0.06**	-0.03	0.01	0.03	0.05
	95% CI	[-0.06, 0.04]	**[-0.11, -0.02]**	[-0.08, 0.03]	[-0.03, 0.05]	[-0.02, 0.07]	[-0.01, 0.10]
FT4	Adjusted Estimate	-0.03	0.24	0.25	-0.11	-0.19	-0.62
	95% CI	[-0.72, 0.67]	[-0.45, 0.94]	[-0.62, 1.12]	[-0.83, 0.58]	[-1.00, 0.58]	[-1.54, 0.26]

All significant associations (p < 0.05) are highlighted in bold. For the analysis log-transformed TSH values are used. Adjusted for smoking and socioeconomic status.

## Discussion

In this study it was found that incidence of thyroid dysfunction in adulthood was not greater in men and women prenatally exposed to the Dutch Famine of 1944-1945 than individuals born shortly before or conceived after the famine. However, mid gestation famine exposure *in utero* affected women’s thyroid function 50 years later, but not men’s. Indeed, amongst mid gestation famine exposed women without overt thyroid dysfunction we found significantly lower TSH values within the normal range. No differences were observed in the FT4 levels of either sex.

The finding that prenatal famine exposure may affect the HPT axis in adulthood is in accordance with previous studies in animals (14, 15) and survivors of the Chinese famine (17, 18), which also found such effects, but did not assess trimester-specific effects or the role of sex therein. Particularly, we found an effect of famine exposure mid-gestation on TSH levels in women. From an ontogenic point of view the second trimester marks the greatest growth of the foetal thyroid during development and is the time that the HPT axis first becomes active (after the 12^th^ week of gestation approximately) (13). Our findings align with previous studies in other organ systems in the DFBC, which found that famine exposure during the second trimester disrupts the normal growth of the lungs and kidneys, which also develop and expand sizably during mid gestation (26, 27).

Previous studies within the DFBC have found that prenatal famine exposure leads to considerable differences in health outcomes, and that timing plays an important role in determining what organ systems are affected (19). Various findings within the DFBC suggest that famine exposure *in utero* affects other biomarkers of metabolism in adulthood as those exposed to famine are more likely to have impaired glucose tolerance (28, 29), a more atherogenic lipid profile (22, 30), and suffer from higher rates of obesity (23, 31), even up to more than threequarters of a century later (19). These effects seem to be sex-specific and timing of exposure during gestation causes significant differences in metabolic phenotypes (23, 30). Those exposed to famine early in gestation, during the time which organogenesis takes place, have a higher chronic disease risk, for example having a higher prevalence of coronary heart disease risk (19, 24). Men exposed to famine in early gestation, but not women, were found to have smaller intracranial and total brain volume compared to unexposed controls (32). Early exposed individuals of both sexes perform worse on selective attention tests (33) and showed signs of premature brain aging in late adulthood (34). Individuals exposed to famine mid gestation, when the respiratory and renal systems undergo rapid growth and development, are more likely to suffer from obstructive airway disease (27) or microalbuminuria (26) in later life. Finally, famine exposure particularly in mid-late gestation is associated with impaired glucose tolerance compared to unexposed individuals (19, 35). This suggests that prenatal famine exposure at the time of the development of vital organs such as the heart, brain, lungs, kidneys, pancreas as well as the HPT axis, and may have long term impacts on the regulation of these physiological systems.

The reduced TSH levels at age 50 in women exposed to famine conditions in mid gestation might be the result of epigenetic alterations within the regulation of the HPT axis. It has been suggested that tissue-specific changes in the epigenetic expression of deiodinase enzymes, which activate and deactivate thyroid hormones, may be a putative mechanism by which such changes in the regulation of the HPT axis can occur (1, 36, 37). Research by Anselmo et al. (2019) on the intergenerational effect of foetal exposure to abnormally high maternal thyroid hormone levels in an Azorean population has shown that even in the third generation, reduced sensitivity to administered thyroid hormones is found (37). They speculated that the pituitary expression of the deiodinase 3 enzyme, which inactivates thyroid hormones by deiodination of the inner ring of both T4 and T3, is increased after exposure to maternal hyperthyroidism during pregnancy (36). The opposite might be true in the context of maternal malnutrition during pregnancy, which has been shown to lead to decreased thyroid hormone levels, specifically T3, compared to well-nourished women (38). Based on this research, we postulate that central feedback suppression in female offspring may be permanently enhanced due to decreased pituitary expression of deiodinase 3, although free T4 levels in the peripheral circulation as measured from fasting blood samples remain unaffected. On the level of the thyroid, prenatal undernutrition in late gestation followed by postnatal overnutrition has been shown to lead to a higher expression of TSH receptors in the thyroid tissue of female sheep, supporting the hypothesis that the thyroid axis is more sensitive to the effects of TSH and thyroid hormones after maternal dietary restrictions during gestation (15). Further research is also needed to elucidate why women but not men experience alterations in the HPT axis due to famine exposure at mid gestation. Interestingly, the second trimester is also when the hypothalamic-pituitary-gonadal (HPG) axis becomes active, which also happens in a sexually dimorphic manner (39). In future research it may be of interest to understand how the sexually dimorphic activation of the HPG axis during this pregnancy trimester interacts with the programming of the HPT axis regulation.

This study has several limitations. Firstly, it is important to consider the general limitations of the DFBC, such as selective fertility in women exposed to the famine of 1944-1945, high rates of perinatal deaths amongst famine exposed pregnancies, and selective survival and participation in the cohort before the follow-up clinics were conducted (19). Secondly, only a single measurement was taken to determine thyroid function, despite evidence that thyroid hormones can fluctuate (40) and are influenced by factors such as seasonality (41), time of the day (42, 43), and menstrual cycle phase for premenopausal women (44). However, all blood samples were taken during the morning. Moreover, only the levels of TSH and FT4 were measured, precluding an estimate of local T4 to T3 conversion, which is known to be affected by starvation *in utero* (38). Thirdly, the thyroid autoantibody levels in this study were slightly lower than reported in more recent studies, but this may be due to more stringent cut-offs being used in the 1990s and/or lower sensitivity of the tests at the time. Finally, iodine status of the mother at the time of gestation remains unknown because of the war circumstances under which the famine took place. There is some evidence from Belgian monks, who persisted during the war by eating tulip bulbs and subsequently developed goitres, suggestive of severe iodine deficiency (45). Severe iodine deficiency among pregnant women (46–48) may impact thyroid function not only in themselves but also in their offspring, through altered thyroid hormone levels in their mothers or decreased transplacental iodine transport.

In this study we showed that the regulation of the HPT axis, which is closely related to metabolic phenotype, may be subtly altered as a result of famine exposure in the second trimester when vital thyroid development and growth take place. However, future research should focus on why the disruption by famine during this period of growth might affect female foetuses differently from male ones. Using the Dutch Famine of 1944-1945 as a historical ‘natural experiment’ this study points towards the existence of subtle sex-specific and trimester-specific effects of severe malnutrition *in utero* on the thyroid physiology of adult survivors.

## Conclusion

In this study of survivors of the Dutch Famine (1944-1945) we found that there are no differences in prevalence of adult thyroid disease at age 50 according to prenatal famine exposure. However, in mid gestation exposed women but not men we find lower TSH levels, which suggest that there may be subtle sex-specific effects of famine exposure during a critical period of thyroid development on the regulation of the HPT axis in adulthood.

## Data Availability Statement

The data that support the findings of this study are available on request from the corresponding author by submitting a formal project outline. The data are not publicly available due to participant privacy restrictions. Code is available upon request from the corresponding author.

## Ethics Statement

The studies involving human participants were reviewed and approved by Medical-Ethical Committee of the Academic Medical Centre of the University of Amsterdam. The patients/participants provided their written informed consent to participate in this study.

## Author Contribution

AR and TR collected the data in 1996-1997. IM is responsible for data management. SK conducted the statistical analyses under supervision of TR, IM, and MF. SK wrote the first draft of the manuscript. All authors contributed to the revisions of the manuscript and approved the final version of the manuscript as submitted.

## Funding

The Dutch famine birth cohort study has been funded by the Medical Research Council (UK), the Diabetes Fonds (Netherlands), Netherlands Heart Foundation grants (NHS2001B087, NHS2007B083; Netherlands), WellBeing (UK), the Diabetes UK, The European Science Foundation (EUROSTRESS-DOME project), the European Commission (Brainage (Seventh Framework Programme Project 279281)), Dynahealth (Horizon 2020 Project 633595), Longitools (Horizon 2020 Project 874739), Well-being (UK, Grant Number NA), the Medical Research Council (UK, Grant Number NA), the Dutch Research Council (NWO Aspasia Project 015014039) and the Academic Medical Centre (Amsterdam, The Netherlands, Grant number NA). SK is supported by a MD/PhD scholarship from the Amsterdam University Medical Centre (Netherlands) and a Moving Forward grant from Amsterdam Reproduction and Development (Netherlands).

## Conflict of Interest

The authors declare that the research was conducted in the absence of any commercial or financial relationships that could be construed as a potential conflict of interest.

## Publisher’s Note

All claims expressed in this article are solely those of the authors and do not necessarily represent those of their affiliated organizations, or those of the publisher, the editors and the reviewers. Any product that may be evaluated in this article, or claim that may be made by its manufacturer, is not guaranteed or endorsed by the publisher.
