# Supplementary material for: Thyroid Function at Age Fifty After Prenatal Famine Exposure in the Dutch Famine Birth Cohort
Source: Front Endocrinol (Lausanne). 2022 Jun 30;13:836245. doi: 10.3389/fendo.2022.836245 (PMC9280834; doi:10.3389/fendo.2022.836245)
Supplement: Supplementary file 1 [file Table_1.docx]

**Supplementary Table 1: Crude thyroid stimulating-hormone (TSH) (geometric mean and interquartile range (IQR)) and free thyroxine (FT4) (mean and standard deviation (SD)) values in individuals exposed to famine without overt thyroid disease (n=675).**

|  | | No in utero exposure | | | Late gestational exposure | | | Mid-gestational exposure | | | Early-gestational exposure | | |
| --- | --- | --- | --- | --- | --- | --- | --- | --- | --- | --- | --- | --- | --- |
|  |  | All (n=407) | Women (n=191) | Men (n=216) | All (n=110) | Women (n=56) | Men (n=54) | All (n=96) | Women (n=55) | Men (n=41) | All (n=62) | Women (n=32) | Men (n=30) |
| TSH (mE/L) | Geometric mean | 1.67 | 1.92 | 1.48 | 1.70 | 1.88 | 1.54 | 1.53 | 1.44 | 1.65 | 1.77 | 1.76 | 1.78 |
|  | IQR | 1.20 | 1.45 | 1.00 | 1.30 | 1.63 | 1.05 | 1.43 | 1.40 | 1.54 | 1.10 | 1.15 | 0.95 |
| FT4 (pmol/L) | Mean | 13.64 | 13.16 | 14.08 | 13.94 | 13.17 | 14.72 | 13.57 | 13.42 | 13.77 | 13.46 | 13.49 | 13.43 |
|  | SD | 2.34 | 2.16 | 2.44 | 4.30 | 2.05 | 5.66 | 2.28 | 2.42 | 2.08 | 2.99 | 3.14 | 2.86 |

**Supplementary Table 2: Differences (and 95% CIs) in thyroid function looking at the effect of being female, overweight, smoking, menopausal, education status, and birthweight using bivariate analyses.**

|  | TSH* (mE/L) | | | FT4 (pmol/L) | | |
| --- | --- | --- | --- | --- | --- | --- |
|  | Estimate | Confidence interval | p-value | Estimate | Confidence interval | p-value |
| Sex female vs male | 0.05 | [0.03, 0.08] | **<0.01** | -0.73 | [-1.08, -0.38] | **<0.01** |
| Menopausal vs premenopausal (women only) | -0.03 | [-0.07, 0.02] | 0.28 | 0.53 | [-0.15, 1.21] | 0.13 |
| Smoking vs non-smoking | -0.04 | [-0.06, 0.01] | **<0.01** | 0.58 | [0.20, 0.95] | **<0.01** |
| Socioeconomic status | 0.001 | [0.00, 0.001] | **0.02** | 0.01 | [-0.01, 0.02] | 0.39 |

*For the analysis log-transformed TSH values are used. All significant associations in bivariate analysis compared to those not in utero exposed are highlighted in bold. Individuals with hypo- or hyperthyroidism, a previous diagnosis of thyroid dysfunction, or currently taking thyroid medication are excluded from this subanalysis.
